# The Omicron BA.2.2.1 subvariant drove the wave of SARS-CoV-2 outbreak in Shanghai during spring 2022

**DOI:** 10.1038/s41421-022-00468-1

**Published:** 2022-09-27

**Authors:** Yun Ling, Gang Lu, Feng Liu, Yun Tan, Xiaoguang Xu, Dong Wei, Jinkun Xu, Shuai Wang, Shuting Yu, Fangying Jiang, Xinxin Zhang, Shuo Chen, Shengyue Wang, Xiaohong Fan, Saijuan Chen

**Affiliations:** 1 Shanghai Public Health Clinical Center, Fudan University, Shanghai, China; 2 Shanghai Institute of Hematology, National Research Center for Translational Medicine, State Key Laboratory of Medical Genomics, Ruijin Hospital Affiliated to Shanghai Jiao Tong University School of Medicine, Shanghai, China; 3 Shanghai Immune Therapy Institute, Renji Hospital, Shanghai Jiao Tong University School of Medicine, Shanghai, China; 4 Department of Infectious Diseases, Research Laboratory of Clinical Virology, National Research Center for Translational Medicine, Ruijin Hospital, Shanghai Jiao Tong University School of Medicine, Shanghai, China

**Keywords:** Structural biology, Immunology

Dear Editor,

As the SARS-CoV-2 virus spreads around the globe, a number of variants of concern (VOCs) have successively dominated geographical incidences of the pandemic of coronavirus disease 2019 (COVID-19)^1,2^. These VOCs are characterized by particular constellations of genetic mutations that are often associated with the change of molecular features of viral proteins promoting viral infectivity and transmissibility, as well as clinical manifestations^3^. Therefore, tracing the origin and spreading patterns of emerging SARS-CoV-2 variants is critical for evaluating and guiding the measures to ameliorate the impact of the virus on public health.

Recently, between late February and early July 2022, the city of Shanghai was hit by a wave of SARS-CoV-2 infections, leading to a total of almost 650,000 laboratory-confirmed cases of COVID-19 and 595 deaths (crude case fatality rate: 0.092%)^4,5^. To identify the SARS-CoV-2 variant(s) causing this outbreak, we used whole-genome targeted sequencing technology to obtain 253 high-quality SARS-CoV-2 genome assemblies from COVID-19 patients diagnosed during the early-to-mid stages of the outbreak (Fig. 1a; Supplementary Table S1). These genomes were then combined with a reference set of SARS-CoV-2 genomes at the Global Initiative on Sharing All Influenza Data (GISAID) database^6^ for an unsupervised phylogeny analysis. The result showed that all our cases clustered into a branch corresponding to the Omicron BA.2.2 lineage, which is characterized by three gained mutations — S:I1221T, ORF1a:T1543I, ORF1a:T4087I — as compared to other strains of SARS-CoV-2 (Fig. 1b; Supplementary Fig. S1). A further analysis using 3,424 BA.2.2 genomes from the GISAID database deposited before April 22nd, 2022 indicated that 239 cases investigated in this study have additionally accumulated two linked mutations — C26789T (M:G89G, synonymous) and A28119G (ORF8:I76V) (Fig. 1c–f; Supplementary Fig. S2) — which characterize BA.2.2.1, a new subvariant of the Omicron BA.2.2 family.

**Fig. 1:**
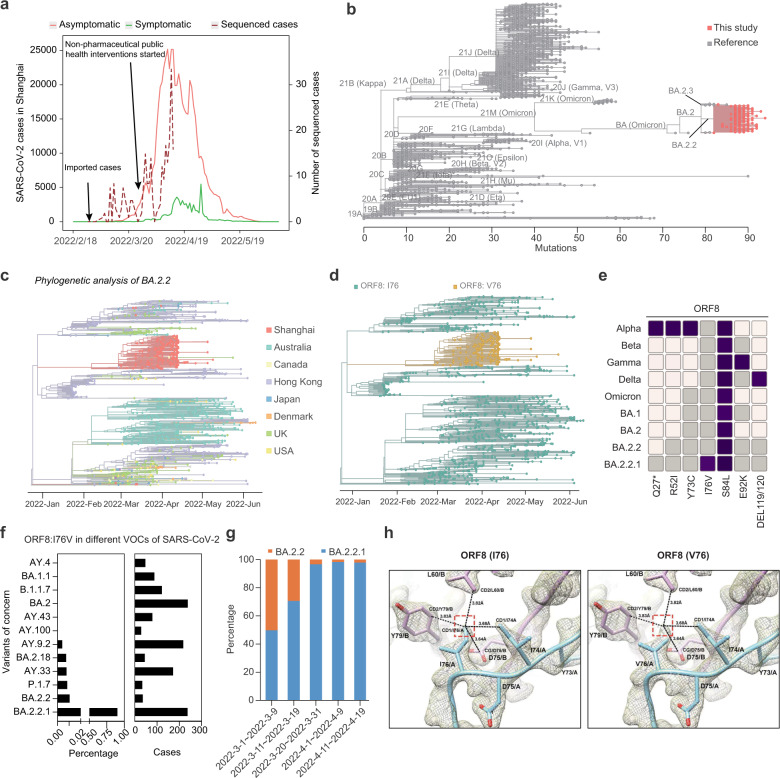
Molecular characterization of the Omicron variant driving the wave of SARS-CoV-2 outbreak in Shanghai during spring 2022. **a** Sample collection times during the recent COVID-2019 outbreak in Shanghai. Total COVID-19 cases are shown in solid lines (red: asymptomatic cases; green: symptomatic cases). Sequenced cases are shown in dashed line. The timeline of the first imported case report and the start of public health interventions are indicated. **b** Phylogenetic analysis of SARS-CoV-2 variants. SARS-CoV-2 reference genomes are shown in gray; 253 SARS-CoV-2 genomes in Shanghai are shown in red. Main SARS-CoV-2 subvariants are indicated. **c** Phylogenetic analysis of SARS-CoV-2 genomes grouped under the BA.2.2 subvariant. Each color indicates the region where each genome was reported from. **d** Phylogenetic tree under the BA.2.2 subvariant as in **c**. The yellow color indicates genomes carrying BA.2.2.1-characteristic mutation: A28119G (ORF8:I76V). **e** Mutation profiles of selected SARS-CoV-2 VOCs. Affected amino acid residues are shown below. Color gradient indicates relative mutation frequencies. Non-mutated sites are colored in gray. **f** The percentages and case numbers of various VOCs of SARS-CoV-2 with the A28119G (ORF8:I76V) mutation. **g** The percentage of BA.2.2.1-infected cases increased over time during the COVID-19 outbreak in Shanghai. **h** I76V at the noncovalent dimerization interface observed in the crystal structure of ORF8 (Protein Data Bank accession code: 7JTL) involving _73_YIDI_76_. ORF8 models are shown as cartoon, and colored in cyan (chain A) and pink (chain B). Electron density (2*F*_O_ − *F*_C_ map, contoured at 1.5σ) is illustrated as gray mesh. van de Waals interactions involving a side-chain carbon (CD1) of I76 are indicated as dashed lines (left panel). A simple I76V mutation is introduced and V76 is refined in real space (right panel).

To determine whether BA.2.2.1 originated from Shanghai, we blasted our BA.2.2.1 sequences against SARS-CoV-2 genomes in the GSIAID database (published as of June 13th, 2022), and found 38 cases of BA.2.2.1. Of them, 6 were reported before the onset of the Shanghai outbreak at mid-late February 2022, whereas the other 32 were reported from the Anhui province of China after March 24th, 2022, when the Shanghai outbreak was already under way. It is noteworthy that the 6 earliest BA.2.2.1 genomes were, respectively, reported from Hong Kong Special Administrative Region (2), Canada (2), Japan (1), and USA (1), and in none of these regions BA.2.2.1 was the dominant circulating SARS-CoV-2 strain (Fig. 1c). Together, these observations suggested that BA.2.2.1 was one minor strain that originated outside of mainland China, which was imported to Shanghai.

On the other hand, while BA.2.2.1 accounted for most of the cases in this study, this was not so during the first 2–3 weeks of the recent outbreak. Between March 1st and March 9th, only half of the cases (5 out of 10) in our study were infected by BA.2.2.1, whereas the other half were infected by BA.2.2 without the linked C26789T (M:G89G, synonymous) and A28119G (ORF8:I76V) mutations (i.e., non-BA.2.2.1 cases, heretofore referred to as BA.2.2). Only later the BA.2.2.1/BA.2.2 ratio gradually increased and BA.2.2.1 became the dominant strain by March 31st (Fig. 1g). This could be the fortuitous result that BA.2.2.1 accidentally outcompeted BA.2.2, for example, by escaping early non-pharmaceutical public health measures that limit their spreading in the community. An alternative, non-excluding, explanation could be that BA.2.2.1 was positively selected over BA.2.2 in the local population in Shanghai. Indeed, based on the published crystal structure of ORF8 (Fig. 1h; Supplementary Fig. S3), the A28119G (ORF8:I76V) mutation undermines a putative dimerization surface that potentially allows ORF8 interactions with host immune response factors^7^. Consistent with this, analysis of a panel of inflammatory factors indicated that IL-5, IL-6, IL-10, IL-12P70 were more significantly activated in the plasma of BA.2.2.1 cases (Supplementary Fig. S4). Moreover, we transduced plasmids expressing wild-type or I76V-mutated ORF8 into A549 cells (a lung epithelial cell line commonly used in the study of respiratory infections) and found that ORF8:I76V differentially upregulated expression of genes encoding the factors related to cytokine-mediated signaling pathways such as IL6, CXCL8, CCL20, and IL11, as well as ACE2, the host receptor of SARS-CoV-2 (Supplementary Fig. S5). These preliminary results suggested that ORF8:I76V might confer a transmission advantage to BA.2.2.1 via promoting expression of inflammatory factors for a more permissive immune response to the virus and/or facilitating the viral entry into the host cells. Of course, the precise molecular function of ORF8:I76V and its relationship with BA.2.2.1 infectivity and host immune responses require further in vitro and/or in vivo studies.

Taken together, our analyses indicate that BA.2.2.1 is a newly emerged Omicron BA.2.2 subvariant that shall be closely monitored given its detrimental role in driving the recent outbreak in Shanghai. It is noteworthy that between early February and late April 2022, Jilin, a province located at northeastern China, also experienced a large wave of SARS-CoV-2 infections, with over 60,000 laboratory-confirmed cases and 3 deaths. Preliminary analysis of 20 genome sequences obtained from the Jilin cases (unpublished) indicates that they were of the Omicron BA.2.1 subvariant. Thus, at least two independent Omicron subvariants were responsible for recent large-scale COVID-19 outbreaks in China in the spring of 2022. It is necessary to continuously monitor all variants, including the more recent BA.4/BA.5 subvariants^8^, in China at population and genome levels and assess their clinical manifestations.

## Supplementary information

Supplementary information

## Data Availability

The data that support the findings of this study are available from the corresponding authors on reasonable request.
