# Supplementary material for: The Omicron BA.2.2.1 subvariant drove the wave of SARS-CoV-2 outbreak in Shanghai during spring 2022
Source: Cell Discov. 2022 Sep 27;8:97. doi: 10.1038/s41421-022-00468-1 (PMC9514698; doi:10.1038/s41421-022-00468-1)
Supplement: Supplementary file 1 — Supplementary information [file 41421_2022_468_MOESM1_ESM.pdf]

Supplementary information

The Omicron BA.2.2.1 subvariant drove the wave of SARS-CoV-2 outbreak in Shanghai during spring 2022

Yun Ling[1,*], Gang Lu[2,*], Feng Liu[2,*], Yun Tan[2,*], Xiaoguang Xu[2,*], Dong Wei[1,*], Jinkun Xu[4], Shuai Wang[2], Shuting Yu[2], Fangying Jiang[2], Xinxin Zhang[3,#], Shuo Chen[4,#], Shengyue Wang[2,#], Xiaohong Fan[1,#], Saijuan Chen[2,#]

Summary

Supplementary information includes methods, one table and five figures.

Methods

Sample collection

This study involved 283 patients, who had tested positive for SARS-CoV-2 RNA and were admitted to the Shanghai Public Health Clinical Center (159 cases) and Shanghai Ruijin Hospital (124 cases) between 1st March and 20th April 2022. Basic demographic, epidemiological and clinical characteristics of enrolled patients are shown in Table 1. This study was approved by the Ethics Committee of Shanghai Public Health Clinical Center (no. 2022-S069-01) in accordance with the Declaration of Helsinki. Informed consent was obtained from enrolled patients.

RNA extraction and SARS-CoV-2 targeting sequencing

Swabs and sputum samples were collected for nucleic acid extraction using an automatic magnetic extraction device and accompanying kit (Shanghai Bio-Germ) and screened using a semi-quantitative RT-PCR kit (Shanghai Bio-Germ) with amplification targeting the ORF1a/b and N genes. The SARS-CoV-2 amplicon libraries were obtained with the Illumina COVIDSeq ARTIC V4.1 kit according to the manufacture's instruction (Illumina). Libraries were sequenced at the Illumina NextSeq 550AR platform (Illumina) according to a PE 150bp protocol in the National Research Center for Translational Medicine (Shanghai). Sequencing reads were trimmed using Trimmomatic (version 0.39)[1] to remove low-quality regions, adaptor sequences and sequencing primers prior to subsequent analysis.

Viral genomic sequence variation calling

Sequencing reads were mapped to reference genome (NCBI Accession: NC_045512.2) with a kmer-based algorithm (kmer-size of 32), valid amplicon targets were evaluated on criteria of ≥50 matched 32-mer. Samples with greater than 85% amplicon coverage passed the QC process and entered downstream analysis. All mapped reads were piled up for assembly consensus and variation call with Illumina DRAGEN COVID Pipeline (v1.1.0). Low-quality regions with read depth below 10 were masked.

Phylogenetic analysis

The assembled genome sequences were evaluated with Nextclade (version 1.11.0) (https://clades.nextstrain.org/) and Pangolin (version 4.0.4)[2]. MAFFT (version 7.490)[3]

was used for multi-sequence alignment after trimming off Ns from both ends of the genome sequences. Nextstrain (version 3.2.4) was used for phylogeny analysis and visualization[4]. The phylogenetic tree was built by IQ-TREE (version 1.6.12) with GTR model. TreeTime (version 0.8.6)[3] was then used for time-resolved phylogeny refinement. The resulting phylogeny tree was visualized using auspice from the Nextstrain package. Bioinformatics analyses were performed on the ASTRA supercomputing platform with DAOS high-performance filesystem in the National Research Center for Translational Medicine (Shanghai).

Quantification of cytokines

The levels of cytokines (IFN-α, IFN-γ, IL-1β, IL-2, IL-4, IL-5, IL-6, IL-8, IL-10, IL-12, IL-17 and TNF) were quantified by the BD™ Cytometric Bead Array (human Th1/Th2/Th17 cytokine kit and Human Inflammatory Cytokine Kit) according to the manufacture's instruction.

Plasmid construction and transfection

Full-length wild-type ORF8 and I76V mutated ORF8 were synthesized by Biosune Biotechnology (Shanghai) and cloned into the pcDNA3.1 plasmid. Plasmids were transfected into A549 cells using the HilyMax Transfection Reagent according to the manufacturer's instructions.

RNA-seq analysis

RNA-seq libraries were constructed with the KAPA RNA HyperPrep Kit according to the manufacturer's instructions. Ribosomal RNAs were removed by the KAPA RiboErase Kit (Human/Mouse/Rat). The libraries were sequenced with the BGI-sequencing platform. Raw reads were mapped to the hg38 reference genome with the STAR methods[5]. Gene read counts were calculated with the Htseq algorithm[6]. Differentially expressed genes were calculated with the DEseq2 package[7].

References

1 Bolger, A. M., Lohse, M. & Usadel, B. Trimmomatic: a flexible trimmer for Illumina sequence data. *Bioinformatics* **30**, 2114-2120, doi:10.1093/bioinformatics/btu170 (2014).

2 O'Toole, A. *et al.* Assignment of epidemiological lineages in an emerging pandemic using the

pangolin tool. *Virus Evol* **7**, veab064, doi:10.1093/ve/veab064 (2021).

3 Sagulenko, P., Puller, V. & Neher, R. A. TreeTime: Maximum-likelihood phylodynamic analysis. *Virus Evol* **4**, vex042, doi:10.1093/ve/vex042 (2018).

4 Hadfield, J. *et al.* Nextstrain: real-time tracking of pathogen evolution. *Bioinformatics* **34**, 4121-4123, doi:10.1093/bioinformatics/bty407 (2018).

5 Dobin, A. *et al.* STAR: ultrafast universal RNA-seq aligner. *Bioinformatics* **29**, 15-21, doi:10.1093/bioinformatics/bts635 (2013).

6 Anders, S., Pyl, P. T. & Huber, W. HTSeq--a Python framework to work with high-throughput sequencing data. *Bioinformatics* **31**, 166-169, doi:10.1093/bioinformatics/btu638 (2015).

7 Love, M. I., Huber, W. & Anders, S. Moderated estimation of fold change and dispersion for RNA-seq data with DESeq2. *Genome Biol* **15**, 550, doi:10.1186/s13059-014-0550-8 (2014).

Supplementary Table

Supplementary Table S1

Table S1 General information of enrolled cases.

	**Entire cohort (n=283)**	**Phylogenetic analysis (n=253)**
**Age**		
≤ 39 --no. (%)	100 (35.34%)	96 (37.94%)
40-49 --no. (%)	42 (14.84%)	30 (11.86%)
50-59 --no. (%)	51 (18.02%)	46 (18.18%)
60-69 --no. (%)	35 (12.37%)	29 (11.46%)
≥ 70 --no. (%)	55 (19.43%)	52 (20.55%)
**Gender**		
Male --no. (%)	144 (50.88%)	132 (52.17%)
Female --no. (%)	139 (49.12%)	121 (47.83%)
**Severe/critical COVID-19**		
Yes --no. (%)	16 (5.65%)	15 (5.93%)
No --no. (%)	267 (94.35%)	238 (94.07%)

Supplementary figures

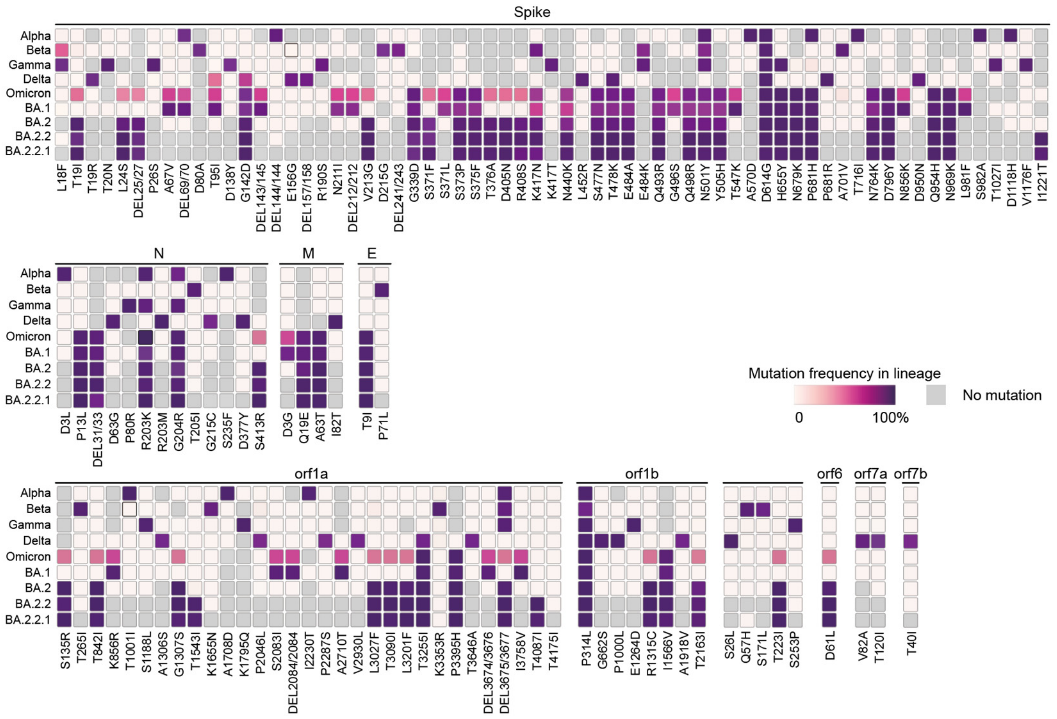

Supplementary Fig. S1. Mutation profiles of selected SARS-CoV-2 VOCs. Affected amino acid residues are shown below. Color gradient indicates relative mutation frequencies. Non-mutated sites are colored in gray.

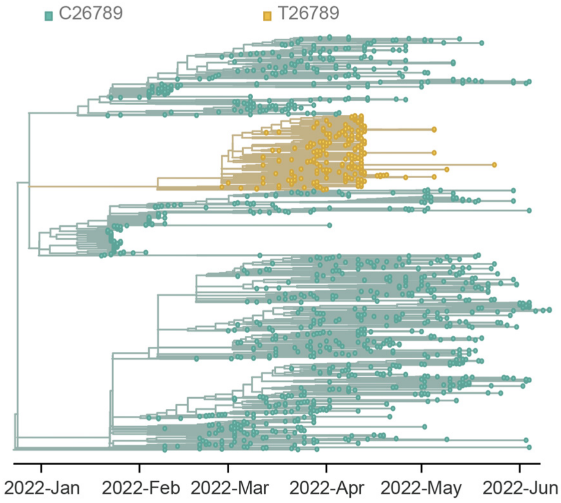

Supplementary Fig. S2. Phylogenetic analysis of SARS-CoV-2 genomes grouped under the BA.2.2 subvariant. The yellow color indicates genomes carrying BA.2.2.1-characteristic mutation: C26789T (M:G89G, synonymous).

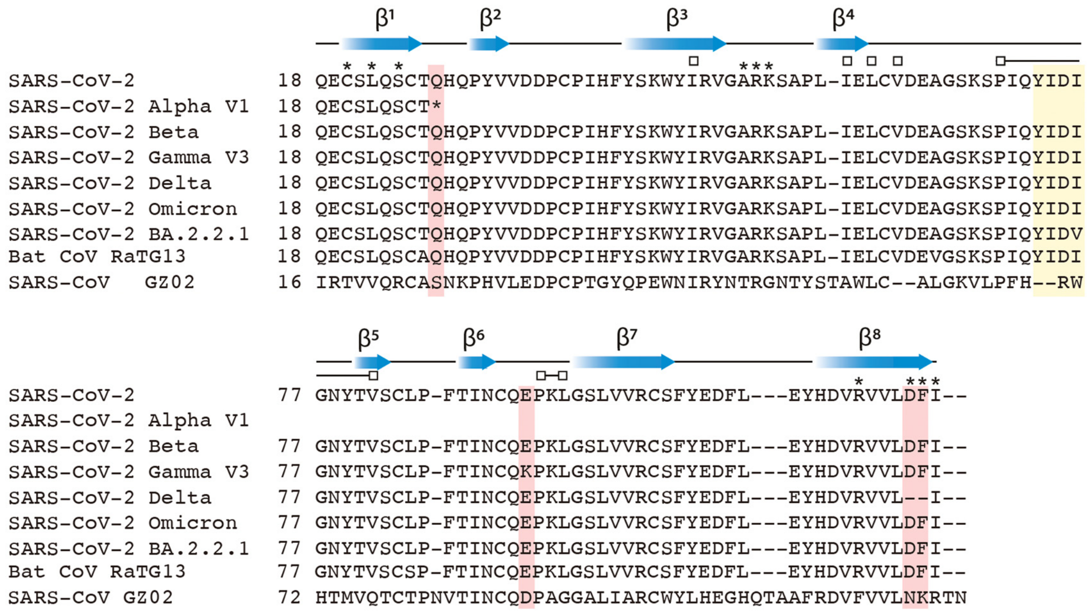

Supplementary Fig. S3. Features of SARS-CoV-2 ORF8. Blue arrows indicate secondary structures of SARS-CoV-2 ORF8. Asterisks indicate residues related to the "covalent" dimer interface. Squares indicate residues related to "non-covalent" dimeric interface.

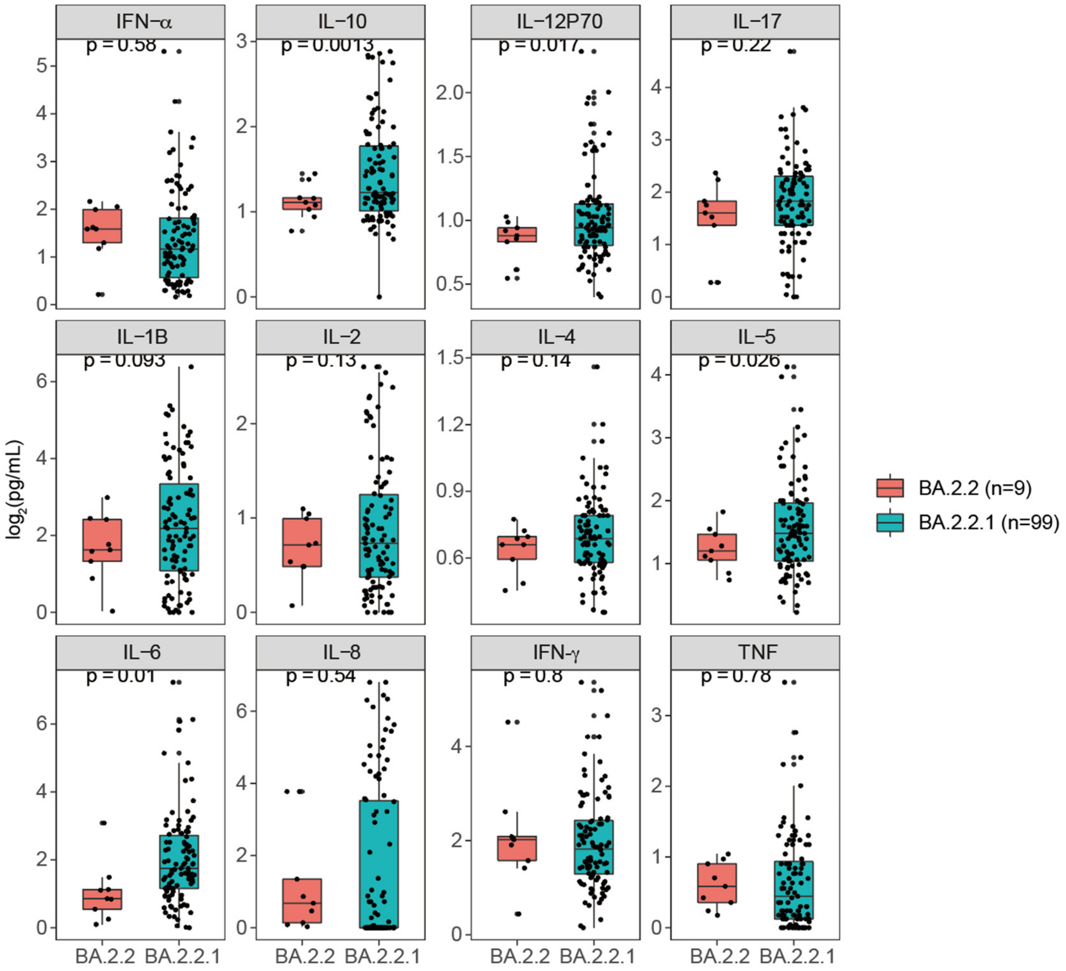

Supplementary Figure S4. Serum levels of indicated inflammatory factors in BA.2.2- and BA.2.2.1-infected individuals at the time of initial diagnosis.

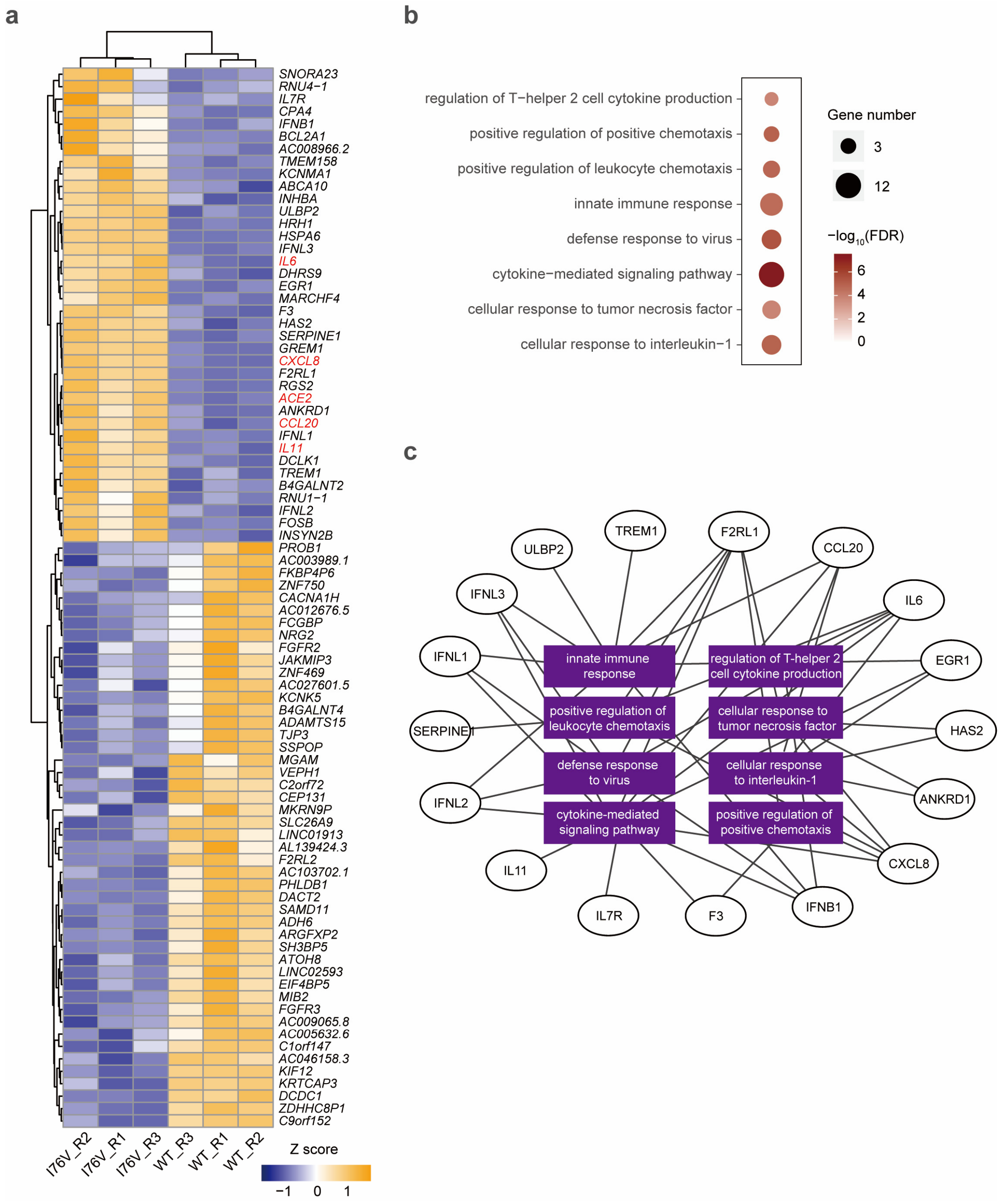

Supplementary Figure S5. Role of I76V mutation on ORF8 function. a Heatmap of differentially expressed genes in A549 cells transfected with plasmid expressing ORF8 (I76V) or wild-type ORF8. **b** Gene ontology analysis of genes differentially upregulated in ORF8 (I76V)-transduced cells. Top-ranked enriched gene ontology terms were shown. Circle sizes indicated enriched gene numbers, and color indicated false discovery rate (FDR). **c** Enriched genes in the top gene ontology terms.
